# The Cross-Level Moderation Effect of Resource-Providing Leadership on the Demands—Work Ability Relationship

**DOI:** 10.3390/ijerph18179084

**Published:** 2021-08-28

**Authors:** Anne Richter, Marta Roczniewska, Carina Loeb, Christiane R. Stempel, Thomas Rigotti

**Affiliations:** 1 Medical Management Center, Department of Learning, Informatics, Management and Ethics, Karolinska Institutet, 171 77 Stockholm, Sweden; marta.roczniewska@ki.se; 2 Center of Research on Cognition and Behaviour, Institute of Psychology, Faculty in Sopot, SWPS University of Social Sciences and Humanities, 81 745 Sopot, Poland; 3 School of Health, Care and Social Welfare, Mälardalen University, Box 883, 721 23 Vasteras, Sweden; carina.loeb@mdh.se; 4 Department of Work & Organizational Psychology, FernUniversität Hagen, 58097 Hagen, Germany; christiane.stempel@fernuni-hagen.de; 5 Department of Work and Organizational Psychology, Johannes Gutenberg University Mainz, 55128 Mainz, Germany; rigotti@uni-mainz.de; 6 Leibniz Institute for Resilience Research, 55122 Mainz, Germany

**Keywords:** emotional demands, workload, role ambiguity, multilevel modeling, psychosocial workplace factors

## Abstract

Employees in female-dominated sectors are exposed to high workloads, emotional job demands, and role ambiguity, and often have insufficient resources to deal with these demands. This imbalance causes strain, threatening employees’ work ability. The aim of this study was to examine whether resource-providing leadership at the workplace level buffers against the negative repercussions of these job demands on work ability. Employees (N = 2383) from 290 work groups across three countries (Germany, Finland, and Sweden) in female-dominated sectors were asked to complete questionnaires in this study. Employees rated their immediate supervisor’s resource-providing leadership and also self-reported their work ability, role ambiguity, workload, and emotional demands. Multilevel modeling was performed to predict individual work ability with job demands as employee-level predictors, and leadership as a group-level predictor. Work ability was poor when employees reported high workloads, high role ambiguity, and high emotional demands. Resource-providing leadership at the group level had a positive impact on employees’ work ability. We observed a cross-level interaction between emotional demands and resource-providing leadership. We conclude that resource-providing leadership buffers against the repercussions of emotional demands for the work ability of employees in female-dominated sectors; however, it is not influential in dealing with workload or role ambiguity.

## 1. Introduction

Work-related stress, sick leave, and work incapacity due to poor mental health have increased [1,2,3,4] and become a serious societal problem worldwide [2,5]. Risk groups for poor work-related mental health can be found in certain sectors [6,7,8], such as the public sector, where human service occupations (e.g., health care, social services, and education) dominate [9,10,11]. These sectors are often referred to as female-dominated sectors. In these sectors, employees are exposed to increased and more specific job demands [12,13,14]. For example, work overload, a lack of formal rewards, and work-life imbalance were found to be particularly problematic demands for health care staff, especially nurses [15]. Additionally, emotional demands are high in human service occupations, where the main work task is to respond to people’s needs [14]. Moreover, a recent report highlighted that certain work demands involving unclear goals, such as a lack of goal clarity or role ambiguity, as well as workload are more prevalent for working women than for working men [7]. Employees in female-dominated sectors often report a lack of resources, which makes it difficult to handle work demands [13,16].

According to the job demands-resources (JD-R) model [17,18], employees’ well-being is dependent on job resources and job demands in their work environment. Whereas job demands (such as workload or role ambiguity) deplete energy and result in job strain and health complaints, job resources (such as social support or autonomy) play a motivating role by stimulating personal growth and goal achievement. Job demands and resources have independent effects through either the *health impairment process* (demands that deplete resources over time) or the *motivational process* (resources that satisfy needs), and demands and resources also interact. According to the JD-R model [17,18], resources buffer against the negative impact of job demands on well-being. Thus, a properly designed work environment requires a balance between demands and resources for employees to deal with those demands. However, the interactions of demands and resources are nuanced [18]. For example, although the negative effects of workloads may be buffered by high autonomy, learning opportunities seem to be less instrumental [19]. Thus, more research is needed to uncover which resources are functional for specific types of demands, which is also emphasized by the compensation principle of the demand-induced strain compensation model [20].

In the workplace, leaders have the ability to shape employees’ work characteristics [21,22,23,24]. Adjusting demands and resources has been identified as one pathway to explain the positive effects of leadership, with other pathways including role modeling and leaders’ capacities to broaden and build the personal resources of their subordinates [25,26,27]. For example, employees with transformational leaders were found to experience fewer job demands and more job resources, which resulted in positive outcomes [28]. However, it is not always possible to limit job demands to achieve a balance between demands and resources; thus, it is important to investigate how resources can be provided to help employees deal with such demands. Research suggests that leaders can be a valuable aid in this regard. For example, in one study, social support, autonomy, and opportunities for growth were specific resources that transformational leaders created and employees utilized to deal with job demands [29,30]. When employees are confronted with increased job strain as a result of taxing job demands, they are more likely to use maladaptive strategies of behavior regulation and cannot escape the loss cycle [31,32]. Therefore, when the job becomes more stressful, stable resources become more important, and positive leadership practices may help employees regulate fatigue and avoid burnout or further losses to their work ability [33].

While transformational leadership has proven to be a valuable leadership style for many employee outcomes, transformational leadership does not specifically focus on creating a resource-oriented work environment. Thus, aligned with the JD-R model, we study resource-providing leadership, which captures the modification of demands and resources as a key leadership task [34,35]. In contrast to general leadership behaviors (e.g., transformational or transactional leadership), resource-providing leadership measures leadership behaviors that are directly focused on improving employees’ health-related job characteristics, such as employee participation or task control. Hence, resource-providing leadership directly operationalizes the notion that leaders affect employee well-being by modifying work characteristics [18,27].

Work ability can be defined as the ability of workers to perform their job taking into account specific work demands on the one hand and resources on the other [36]. Thus, representing the notion of a proper balance between individuals’ perception of their work demands and resources, work ability could be a proximal outcome of leaders’ actions related to demands and resources at the workplace. As an employee’s greatest asset [37], work ability has frequently been studied, particularly in Nordic countries, as a predictor of long-term sickness absence and as a risk factor for early disability pensions [38]. A time-lagged study among employees of two organizations showed that low work engagement was related to low work ability beyond known health behaviors and psychosocial work characteristics [39]. This points to the relevance of job resources for preserving work ability by boosting work engagement. Indeed, a recent study by Boelhouwer and colleagues tested four types of job resources and their roles in work engagement and work ability. Autonomy and supportive leadership (but not colleague support) demonstrated both a direct and buffering role in these two outcomes [40].

**Figure 1:**
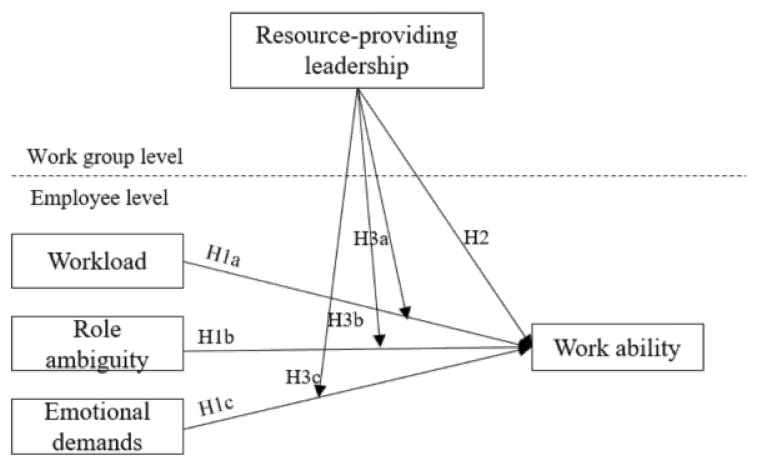
The conceptual model of employee-level job demands and work group-level resource-providing leadership predicting work ability.

The overall aim of the present study is to investigate how specific demands that are prevalent in female-dominated sectors relate to work ability and how resource-providing leadership at the group level affects this relationship (see Figure 1 for an overview of the conceptual model). In line with existing research [7,14,15], this study investigates workload, role ambiguity, and emotional demands, presenting demands that might be particularly relevant for employees in female-dominated sectors. More specifically, the following hypotheses are investigated:

**Hypothesis** **1a–c.** *The individual-level job demands of workload (a), role ambiguity (b), and emotional demands (c) are negatively related to work ability.*

**Hypothesis** **2.** *Group-level resource-providing leadership is positively related to work ability.*

**Hypothesis** **3a–c.** *Group-level resource-providing leadership moderates the relationships between individual-level predictors (workload (a), role ambiguity (b) and emotional demands (c)) and work ability such that the individual-level relationships weaken as resource-providing leadership at the group level increases.*

## 2. Materials and Methods

### 2.1. Participants and Procedure

The current study was part of a larger research project on leadership and health in Finland, Germany, and Sweden [41]. Participants were approached via the human resources department or executive management of their organizations. In all cases, employee representatives were included in the process of obtaining their consent. Data were collected via online and paper-pencil questionnaires, and participants were assured of the confidential and anonymous treatment of their information. The proximity between leaders and employees (in terms of both location and hierarchy) was an important selection criterion in the sampling strategy. In our sample, leaders always directly supervised their work groups, and work group members reported (directly) only to this manager. In total, 3336 questionnaires were distributed, and usable data (i.e., when employees completed all scales of interest) were obtained from 2383 employees (71% response rate) nested in 290 work groups. Participants were located in three countries: Germany (*n* = 1363; 57%), Finland (*n* = 586; 25%), and Sweden (*n* = 434; 18%). Over half of the study sample was employed in a public sector organization (*n* = 1352; 57%), and most had a permanent contract (94%). All jobs from the examined sectors—finance (Germany), public administration, healthcare, service, and education (Finland, Germany, and Sweden)—were characterized by high service demands and a customer orientation and required regular interaction and exchange among work group members and their immediate manager. Reflecting the gender structure of the female-dominated sectors that we investigated, the majority of the sample were women (*n* = 1849; 78%). On average, the study participants were 43.63 years old (*SD* = 10.64). Their average work tenure at the organization was 14.65 years (*SD* = 9.90), whereas their average tenure in their work group was 7.31 years (*SD* = 7.67). Work hours per week ranged from 10 to 65, with an average of 40.

### 2.2. Measures

For all scales, the distinctiveness of the subscales was previously assessed using confirmatory factor analysis in this sample, and appropriate psychometric properties (including measurement invariance between the three different language versions) were found [41]. In addition to using the scales listed below, we gathered demographic data such as age, gender, and tenure.

*Workload.* Workload was measured with five items from the Quantitative Workload inventory [42] using a 5-point Likert-type scale ranging from 1 (*very seldom or never*) to 5 (*very often or always*). Two sample items are “How often does your job require you to work very fast?” and “How often does your job require you to work very hard?” Cronbach’s alpha for reliability was 0.79.

*Role ambiguity*. Role ambiguity was measured with three items from the Copenhagen Psychosocial Questionnaire (COPSOQ II) [43] using a 5-point Likert-type scale ranging from 1 (*to a very small extent*) to 5 (*to a very large extent*). A sample question is “Does your work have clear objectives? [reversed]”. Cronbach’s alpha for reliability was 0.74.

*Emotional demands*. Emotional demands were measured with four items from the COPSOQ II [43]. Two of the items were measured on a 5-point Likert-type scale ranging from 1 (very seldom or never) to 5 (very often or always). A sample item is “Does your work put you in emotionally disturbing situations?” The two other items were measured on a 5-point Likert-type scale ranging from 1 (*to a very small extent*) to 5 *(to a very large extent*). A sample question is “Is your work emotionally demanding?” Cronbach’s alpha for reliability was 0.85.

*Resource-providing leadership.* Resource-providing leadership was measured with 10 items covering the three areas of task control, participation, and conflict management from the Health- and Development-Promoting Leadership Behavior Questionnaire [34,44]. Subscales from the resource-providing subfactor were chosen, and here, we identified those which fit the demands and context of the female-dominated sector best. Specifically, we aimed at resources that best match the demands we investigated. Providing task control has been shown to mitigate the negative effects of workloads, [45,46], participation seems suitable to negatively affect role ambiguity [47], and conflict management may be especially helpful in dealing with emotional demands [48,49]. Employees rated their immediate manager on a 5-point Likert-type scale ranging from 1 (*strongly disagree*) to 5 (*strongly agree*). Sample items are “My immediate superior allows me to decide for myself how I organize my tasks” (task control), “My immediate superior includes me in decisions that affect my work or workplace environment” (participation), and “My immediate superior searches for solutions to conflicts with those involved” (conflict management). Cronbach’s alpha for reliability was 0.86. This variable was used as a group (i.e., level 2) predictor, and therefore had to be aggregated. Aggregation was justified based on ICC(1) = 0.24 and ICC (2) = 0.72, as well as a mean r_wg_ of 0.83.

*Work ability.* Work ability was assessed with one item from the Work Ability Index [36,50] measuring perceived work ability in relation to the demands of the job (“What is your work ability like in relation to the demands of your job?”). The item was rated on a 10-point Likert-type scale ranging from 0 (*bad*) to 10 (*good*).

### 2.3. Analytical Strategy

We applied hierarchical linear modeling (HLM) analyses to test all hypotheses using HLM 7 [51]. The HLM approach was warranted because the structure of the data was nested (employees nested within work groups, work groups nested within countries), which violated assumptions on the non-interdependence of the data, affecting standard error estimations [52]. We calculated ICC(1) at the work group and country levels using a formula presented in Hox et al. [52]. This formula refers to ICC values as the expected correlation of work ability between the observations of two randomly chosen persons in the same work group (work group-level ICC) or country (country-level ICC). The ICC(1) at the work group level was 0.15, and the ICC(1) at the country level was 0.07. These estimates suggested the need to control for the nesting effect and warranted the use of HLM.

To investigate the direction of the cultural differences, a one-way analysis of variance was conducted at the individual level for all variables. Country was used as a factor with three values. Dunnet C-corrected pairwise comparisons showed significantly lower work ability among German (M = 7.60) than Swedish (M = 8.72) or Finnish (M = 8.54) participants, with no differences between the latter two. Moreover, Germans reported significantly lower emotional demands (M = 3.08) than Finnish participants (M = 3.28), who showed lower demands than Swedish employees (M = 3.52). For resource-providing leadership, Germans (M = 3.55) reported significantly lower levels than Finnish or Swedish employees (M = 3.89 and M = 3.85, respectively). No differences regarding role ambiguity and workload were detected.

Moreover, the model comprised the effect of three job demands at the individual level and the cross-level effect of resource-providing leadership at the work group level (for the conceptual model, see Figure 1) and thus required HLM.

We performed five consecutive models following the procedure delineated in Hox et al. [52]. First, in Model 1, we estimated the null model (with no predictors involved) to decompose the total variance of work ability into three terms (employee, work group and country levels) to benchmark the model fit and explained variance. In Model 2, we added Level 1 predictors (workload, role ambiguity, and emotional demands) to estimate the fixed effects at the individual level. Model 2 tested Hypotheses 1a–c. Because age is likely to affect work ability [53], we added this variable in Model 2 as a control. In the third model (intercept-as-outcome), the intercept estimates derived from the Level 1 analysis were regressed on resource-providing leadership to test whether the latter accounted for the between-group variance in work ability. More specifically, this model tested the cross-level effect of resource-providing leadership at the group level on individual work ability (Hypothesis 2). In Model 4, we estimated whether the relationships between job demands and work ability varied between work groups. The slope of workload, role ambiguity, and emotional demands were tested for randomness in separate models to eliminate the number of random parameters tested at once [52]. We report the model where the variance of the slopes was significant, indicating that the slope differed depending on the work group. Finally, the fifth model tested for cross-level moderating effects that would reveal whether the relationships between any of the job demands and work ability at the individual level varied depending on resource-providing leadership at the work group level (slope-as-outcome). Thus, this final model tested Hypotheses 3a–c. To analyze the effects at various values of the moderator, we used the 16th and 84th percentiles of resource-providing leadership, as Hayes [54] recommends probing at percentiles to guarantee that the probed points are always within the observed range of the data when the distribution diverts from normal. If the distribution of the data is normal, the 16th and 84th percentiles correspond to ±1 SD from the mean.

Following Enders and Tofighi [55], we group-mean-centered Level 1 predictors and grand-mean centered the Level 2 predictor. Group-mean centering is recommended for Level 1 predictors when investigating cross-level interactions, as it allows distinguishing cross-level interaction from between-group interaction [56]. Simultaneously, grand-mean centering is a better choice for scaling for Level 2 predictors because it helps reduce the covariance between intercepts and slopes, thereby reducing potential problems associated with multicollinearity.

## 3. Results

Table 1 presents the descriptive statistics of the study variables and simple correlations, whereas Table 2 presents the results of the multilevel analysis.

In the intercept-only model (Model 1), the variance components of work ability were significant at both the work group and country levels (*p*s < 0.001). In Model 2, where we entered group-mean centered Level 1 job demands (role ambiguity, workload, and emotional demands) and controlled for age, the overall model fit improved, that is, deviance decreased: ∆ D (4) = 184.04, *p* < 0.001. As expected, when we controlled for age, work ability was negatively predicted by workload (γ = −0.35, SE = 0.06, *p* < 0.001), role ambiguity (γ = –0.52, SE = 0.06, *p* < 0.001), and emotional demands (γ = –0.35, SE = 0.05, *p* < 0.001). Supporting Hypothesis 1a, as workload increased, work ability decreased. In line with Hypothesis 1b, employees who experienced more role ambiguity within their group reported poorer work ability. Finally, as predicted by Hypothesis 1c, employees who expressed having more emotional demands reported poorer work ability.

When we added resource-providing leadership as a Level 2 predictor (Model 3), the model fit significantly improved: ∆ D (1) = 36.62, *p* < 0.001. Resource-providing leadership was positively related to work ability (γ = 0.68, SE = 0.11, *p* < 0.001). In work groups led by leaders rated as more resource-providing, employees reported better work ability. This result supports Hypothesis 2.

We performed three model 4 versions, where we estimated whether the relationships between each of the job demands and work ability varied between work groups. Only the random slope of emotional demands was significant (Hypothesis 3c), and the other models did not converge under the conventional number of 100 iterations for the random slopes of workload (Hypothesis 3a) and role ambiguity (Hypothesis 3b). Model 4, with the random slope of emotional demands and fixed slopes of workload and role ambiguity, had a significantly better fit than Model 3: ∆ D (2) = 20.95, *p* < 0.001. The significant variance component of the random slope of emotional demands (*p* = 0.010) indicated that the negative relation between the level of emotional demands and work ability differed depending on the work group.

**Figure 2:**
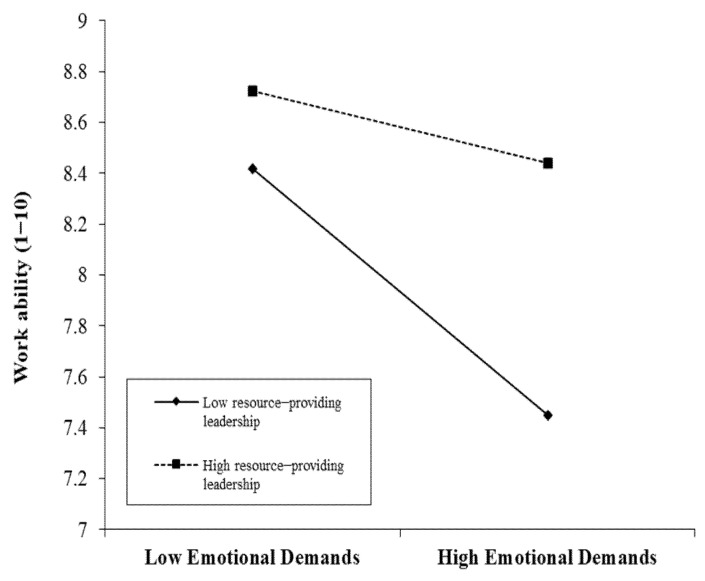
Prediction of work ability by emotional demands (Level 1 and group−mean centered) as a function of resource−providing leadership (Level 2).

Thus, in the final model, we tested whether the randomness of the emotional demands–work ability slope could be explained by resource-providing leadership. Adding this cross-level interaction significantly improved the model fit: ∆ D (1) = 9.61, *p* = 0.002. Figure 2 demonstrates the buffering role of resource-providing leadership in the link between emotional demands and work ability (γ = 0.39, SE = 0.13, *p* = 0.002). The lines represent the slopes for low (16th percentile) and high (84th percentile) levels of resource-providing leadership.

Simple slopes analysis revealed that in groups where leaders were low in resource-providing leadership, there was a negative link between emotional demands and work ability (simple slope = −0.48 (0.05), *p* < 0.001). In those work groups, the greater employees’ emotional demands, the lower their work ability was. For high values of resource-providing leadership, this relationship was reduced (simple slope = −0.14 (0.07), *p* = 0.051). In work groups led by high resource-providing leaders, emotional demands were not significantly linked with work ability. The difference between high and low resource-providing leadership was most pronounced for high values of emotional demands, where low resource-providing leadership was linked with a substantial reduction in work ability compared to high resource-providing leadership (simple slope = 1.13 (0.18), *p* < 0.001). When emotional demands were low, the difference was smaller, but the work ability of individual employees was still better in work groups led by high resource-providing leaders than in work groups led by low resource-providing leaders (simple slope = 0.35 (0.17), *p* = 0.04).

## 4. Discussion

This study aimed to investigate how workload, role ambiguity, and emotional demands, which all present specific demands that are prevalent in female-dominated sectors [7], decrease work ability and to examine the extent to which group-level resource-providing leadership buffers against these negative effects. In line with the health impairment process proposed by the JD-R model, the findings show that workload, role ambiguity, and emotional demands were negatively associated with work ability. We propose that these factors should be included in the assessment of risk factors in the psychosocial work environment in female-dominated sectors. Additionally, congruent with the motivational process in the JD-R model, team-shared perceptions of resource-providing leadership were positively related to work ability. This result is congruent with recent findings from the population-based prospective MONICA/KORA study, which demonstrated that people who did not feel supported by their supervisor had a significantly higher risk for suboptimal health ten years later than those who did [57].

In this study, we assumed that leaders who lead in a resource-providing way act as a buffer between work-related demands and employee work ability. The results showed that this buffer effect was visible for one of the three risk factors. Specifically, resource-providing leadership protected against the negative effects of emotional demands on work ability. However, we did not observe such effects for workload or role ambiguity. There are several possible reasons for these mixed findings. Resource-providing leadership might be better suited to help employees deal with the repercussions of emotional demands on work ability than to help employees deal with other job demands we investigated. For example, servant leadership at cafés and coffee shops buffers the negative impact of customer mistreatment on employee outcomes [58]. When employees have to handle difficult situations with customers or colleagues, they might perceive their manager’s efforts to provide them with resources as particularly helpful and supportive. Thus, these resources may help them deal with demands from other people. Workload, on the other hand, might be affected by factors that a line manager does not have the power to address. This demand may depend more on contextual factors, such as the number of patients who seek care at a particular time or the contracts that an organization has signed, which set the target for employees. Additionally, positive leadership may be more relevant for workload when tested on a day-to-day basis: a diary study among teachers demonstrated a buffering role of transformational leadership on the link between workload and work engagement [59]. Alternatively, a different type of leadership behavior was shown to be relevant for increasing demands among police officers, such as health-promoting leadership [60]. When leaders show attention and sensitivity to issues of health or health-impairing work conditions (e.g., noticing when employees need a break), this approach has a buffering effect on employee burnout. Thus, workload may require more immediate or different types of behaviors than what is offered by resource-providing leadership. Even though role ambiguity was associated with work ability, employees indicated a low level of role ambiguity in general. Hence, role ambiguity was not perceived as problematic in this sample, in contrast to other literature on role ambiguity [7]. Under these circumstances, a buffering effect might be neither relevant nor easy to find.

This study makes several important contributions. First, we contribute to theory development by showing that resources and demands need to be matched. In this study, resource-providing leadership was found to be a buffer only for emotional demands and not for the other demands under investigation. The results thus point to the need to find a match between demands and resources that are instrumental in coping with these demands. With these results, we answer the call for a more nuanced analysis of the interactions between job demands and job resources [19]. Second, this study makes an important practical contribution by showing that workplace-level factors such as leadership are important and affect individual-level outcomes such as work ability. These findings are in line with current recommendations that organizational interventions that aim to change how work is designed, organized, and/or managed [61] are more effective than individual interventions that aim to address the primary causes of poor health [62,63]. Providing training to leaders can be an example of an effective organizational intervention [64,65,66]. Not only do leaders themselves benefit from these trainings, but most importantly, improved leadership skills affect leaders’ subordinates [67]. Since training leaders are an organizational intervention that can have widespread positive effects, establishing a training program could be a promising way to help leaders address specific work-related needs. In particular, intervention programs that target factors at the team level and are participatory seem promising [66,68,69,70]. Leaders who actively involve their employees in shaping the work conditions could facilitate more sustainable results because the focus is beyond individual interactions. Thus, these findings advance the research on female-dominated sectors in highlighting the importance of shared perceptions of workplace-level resources such as resource-providing leadership to promote sustainable employment.

### Strengths and Limitations

There are several strengths to this study. First, we addressed the nested data structure of our sample by applying a multilevel approach. This design allows us to investigate the effects that are beyond individual perceptions. Namely, when individual employee ratings are aggregated to show the group-level perception of leaders’ behaviors, it may be considered a more objective assessment of his or her skills. Furthermore, treating leadership as a group-level variable enabled us to compare how these differences in managers’ behaviors influence processes observed at the individual level (here, links between individual job demands and work ability). Such analyses are vital to discover how processes may depend on organizational contexts. Second, we drew on a large sample from multiple service-oriented organizations in three countries; hence, we found robust results that can be generalized to this sector.

There are several limitations to this study. First, the cross-sectional nature of our sample does not allow for any inferences about causality. It is possible that low work ability resulting from, for example, functional disabilities or health problems results in employees perceiving their job demands as more intense, which would indicate reversed causality. Therefore, future studies should validate our results using longitudinal data on the time-lagged effects for work ability. Second, we investigated leadership as a possible group-level factor that could buffer job demands that are particularly important for female-dominated sectors. Future studies could investigate other factors relevant to these job demands to build strong empirical evidence that can become the basis for organizational interventions in female-dominated sectors. One such factor could be social job resources, such as teamwork, which seems especially relevant in female-dominated environments [71]. Moreover, investigating other sectors requires a conscious choice of demands that might be particularly important in those sectors. Finally, in this study, we focused on resources at the work unit level; however, other hierarchical levels, such as the team or department levels, might also be interesting to investigate, as the decision latitude for job demands and resources varies across levels [72].

## 5. Conclusions

In summary, we find that resource-providing leadership is a way to help employees from the female-dominated sector dealing with certain job demands. While we studied three sector-relevant demands (i.e., workload, role ambiguity and emotional demands) and all three as well as resource-providing leadership were relevant for employees’ work ability, resource-providing leadership buffered only against the repercussions of emotional demands. Our study makes important contributions to the literature on resources and demands and particularly the matching of these to foster occupational health. Moreover, our results also indicate that leadership development focusing on resource-providing leadership might be a relevant avenue for the future organizational interventions. Furthermore, future research should investigate other demands and resources at different organizational levels to further our understanding of the match between demands and resources and to provide a basis for evidence-based organizational interventions.

## Figures and Tables

**Table 1:** Descriptive statistics of the study variables and sample with 95% CI.

	M (SD)	WA	RA	W	ED	RPL
Work ability (WA)	8.04 (1.80)	—	−0.08 *** [−0.12; −0.04]	−0.22 *** [−0.26; −0.18]	−0.16 *** [−0.20; −0.12]	0.23 *** [0.19; 0.27]
Role ambiguity (RA)	1.92 (0.63)	0.15 * [0.03; 0.26]	—	−0.12 *** [−0.16; −0.08]	−0.15 *** [−0.19; −0.11]	−0.07 *** [−0.11; −0.03]
Workload (W)	3.62 (0.65)	−0.33 *** [−0.43; −0.22]	−0.27 *** [−0.37; −0.15]	—	0.38 *** [0.34; 0.41]	−0.04 * [−0.08; −0.00]
Emotional demands (ED)	3.21 (0.93)	−0.24 *** [−0.25; −0.13]	−0.34 *** [−0.44; −0.24]	0.44 *** [0.34; 0.53]	—	0.12 *** [0.08; 0.16]
Resource-providing leadership (RPL)	3.69 (0.34)	0.44 *** [0.34; 0.53]	−0.12 * [−0.23; −0.00]	−0.04 [−0.15; 0.08]	0.13 * [0.01; 0.24]	—

Notes: Correlations at the employee level (*N* = 2383) are displayed above the diagonal. Correlations at the work unit level (*N* = 290) are displayed below the diagonal. The correlational analyses do not account for the nested structure of the data. *** *p* <.001, * *p* < 0.05.

**Table 2:** Multilevel analysis predicting work ability from job demands (Level 1) and resource-providing leadership (Level 2), controlling for country-level variance (Level 3).

	Model		Model		Model		Model		Model	
	1		2		3		4		5	
***Fixed part***	Est.	SE	Est.	SE	Est.	SE	Est.	SE	Est.	SE
Intercept	8.30 **	0.29	8.30 **	0.29	8.26	0.23	8.22	0.20	8.22	0.20
**Level 1−Employee**										
Age			–0.01 **	0.00	–0.01 **	0.00	–0.01 **	0.00	–0.01 **	0.00
Role ambiguity (RA)			–0.52 ***	0.06	–0.52 ***	0.06	–0.52 ***	0.06	–0.51 ***	0.06
Workload (W)			–0.35 ***	0.06	–0.35 ***	0.06	–0.36 ***	0.06	–0.36 ***	0.06
Emotional demands (ED)			–0.35 ***	0.05	–0.35 ***	0.05	–0.36 ***	0.05	–0.34 ***	0.06
**Level 2−Work group**										
Resource-providing leadership (RPL)					0.68 ***	0.11	0.65 ***	0.11	0.72 ***	0.11
Cross-level interaction										
ED × RPL									0.39 **	0.13
***Random part***										
Variance decomposition										
Employee	2.77		2.54		2.53		2.44		2.44	
Work group	0.23		0.26		0.20		0.22		0.22	
Country	0.24		0.24		0.15		0.11		0.12	
Random slope of ED							0.16		0.13	
***Model fit***										
Deviance (D)	9345.39		9161.35		9124.73		9103.78		9094.17	
Number of estimated parameters	4		8		9		11		12	
∆ D (M_n−1_)			184.04 ***		36.62 ***		20.95 ***		9.61 ***	
∆ parameters (M_n−1_)			4		1		2		1	

Notes: N_employees_ = 2383, N_work groups_ = 290. Est. = parameter estimate. M_n_ = model number. Age, role ambiguity, workload, and emotional demands are group-mean centered. Resource-providing leadership is grand-mean centered. *** *p* < 0.001, ** *p* < 0.01, * *p* < 0.05.

## Data Availability

Data supporting these findings can be provided upon request.
